# Takotsubo Cardiomyopathy After Atrioventricular Synchronous Leadless Pacemaker Implantation for Complete Atrioventricular Block

**DOI:** 10.1016/j.cjco.2025.08.007

**Published:** 2025-08-26

**Authors:** Koji Sudo, Kenji Kuroki, Chisa Asahina, Akira Sato

**Affiliations:** Department of Cardiovascular Medicine, Faculty of Medicine, University of Yamanashi, Chuo, Japan

**Keywords:** Complete atrioventricular block, leadless pacemaker, Takotsubo cardiomyopathy

**An 84-year-old woman with complete atrioventricular block and heart failure underwent atrioventricular synchronous leadless pacemaker (Micra AV) implantation under conscious sedation. Despite a successful procedure without immediate complications, routine transthoracic echocardiography revealed left ventricular apical akinesis with basal hyperkinesis, consistent with Takotsubo cardiomyopathy. Multimodality evaluation confirmed the diagnosis, and cardiac function gradually recovered. This rare case highlights stress-induced cardiomyopathy following leadless pacemaker implantation.**

An 84-year-old woman was referred to our hospital for complete atrioventricular (AV) block with heart failure. The patient had severe dementia and had a small build (height, 143 cm; weight, 45 kg). Transthoracic echocardiography on admission showed normal left ventricular (LV) systolic function. We performed AV synchronous leadless pacemaker (LP) (Micra AV, Medtronic, Minneapolis, MN) implantation with the patient under conscious sedation with dexmedetomidine. The patient did not appear anxious before the procedure, likely due to severe dementia. The LP was successfully deployed at the lower septum of the right ventricle (Fig. 1A) without any complications. The patient remained properly relaxed during and after the procedure and did not present with clinical symptoms the day after the procedure; however, routine transthoracic echocardiography revealed akinesis in the LV apex and hyperkinesis in the basal left ventricle with a LV systolic function of 54% (Fig. 1B; Video 1 , view video online). Coronary computed tomography imaging showed no significant stenosis of the coronary arteries, but myocardial scintigraphy with dual nuclides showed a mismatch between ^201^thallium and ^123^I-beta-methyl-iodophenyl pentadecanoic acid (Fig. 1C). These multimodality findings suggest Takotsubo cardiomyopathy. Transthoracic echocardiography performed 30 days after the procedure revealed that LV systolic function had improved to 68% (Fig. 1D; Video 2 , view video online). In ventricular pacing, giant negative T waves and QT prolongation were observed in the anterior precordial leads; however, these abnormalities improved over time in a 12-lead electrocardiogram (Fig. 1E). In our case, the onset factors were thought to be mechanical stress caused during cardiac implantable electronic device implantation^1^^,^^2^ or physical stress caused by pain during the insertion of a 27-Fr LP delivery catheter into the femoral vein. One year after implantation, the pacing threshold remained stable while atrioventricular synchrony pacing was maintained. Although the patient was asymptomatic, multimodality images led to a diagnosis of Takotsubo cardiomyopathy after LP implantation.

**Novel Teaching Points**

• Takotsubo cardiomyopathy (TCM) is a rare disease triggered by mechanical or physical stress.
• Myocardial scintigraphy with _201_thallium and _123_I-beta-methy-iodophenyl pentadecanoic acid in the acute phase is useful for the appropriate diagnosis of TCM.
• TCM should be considered a potential complication of leadless pacemaker implantation.

**Figure 1:**
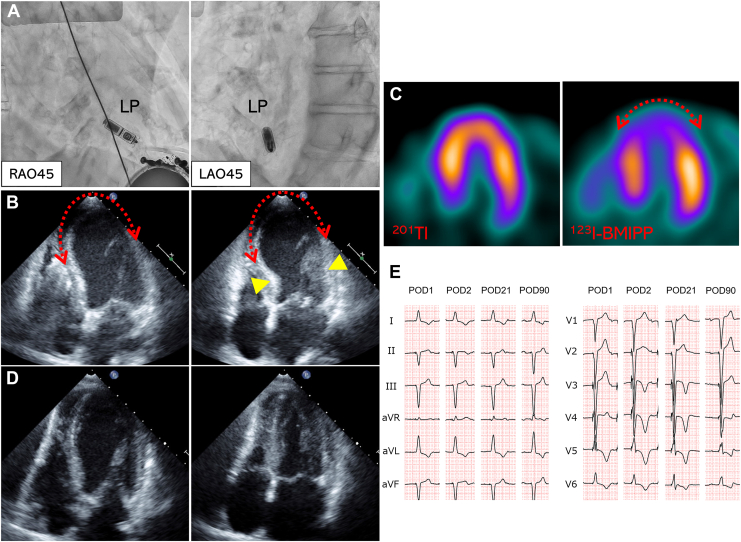
(**A**) Radiographic images in right anterior oblique (RAO) and left anterior oblique (LAO) views after atrioventricular synchronous leadless pacemaker (LP) implantation. (**B**) Transthoracic echocardiography on the day after the procedure. The left ventricular apex shows akinesis (**red dotted arrows**) and the basal left ventricle shows hyperkinesis (**yellow arrowheads**). (**C**) Dual scintigraphy showing a mismatch between ^201^thallium (^201^Tl) and ^123^I-beta-methyl-iodophenyl pentadecanoic acid (^123^I-BMIPP) (**red dotted arrow**). (**D**) Transthoracic echocardiography on postoperative day 30, showing normal left ventricular systolic function. (**E**) Changes in 12-lead electrocardiography findings over time. POD, postoperative day.

## Supplementary Material

Video 1 Transthoracic echocardiography revealed akinesis in the LV apex and hyperkinesis in the basal LV the day after the procedure.

Video 2 Transthoracic echocardiography performed 30-days after the procedure revealed that LV systolic function had improved.
